# Keratan Sulfate Is a Multifunctional Brain Glycosaminoglycan With Instructive Capabilities

**DOI:** 10.1111/jnc.70208

**Published:** 2025-08-27

**Authors:** James Melrose

**Affiliations:** ^1^ Raymond Purves Bone and Joint Research Laboratories, Kolling Institute of Medical Research, Faculty of Medicine and Health The University of Sydney and the Northern Sydney Local Health District, Royal North Shore Hospital Sydney New South Wales Australia; ^2^ Graduate School of Biomedical Engineering, Faculty of Engineering University of New South Wales Sydney New South Wales Australia

**Keywords:** axonal guidance, KS, KS‐proteoglycans, neuronal cell regulation, perineuronal nets

## Abstract

Keratan sulfate (KS) is a glycosaminoglycan (GAG) with unique functions including electroconductive properties that support neurotransmission. KS‐proteoglycans contribute to tissue stabilization and functional organization, and have diverse interactive properties with cytokines, growth factors, morphogens, neuregulatory proteins, and neuron receptors that control the formation and function of neural networks. As side chain components of a diverse range of brain proteoglycans, KS assists neural development and axonal guidance, storage and transport of neurotransmitters in synaptic vesicles, neurotransduction, synaptic plasticity, cognition, and memory in perineuronal nets, neuronal proliferation, and differentiation. KS is thus a multifunctional instructive brain GAG with essential roles to play in brain function and homeostasis, with instructive roles in the assembly and repair of functional network structures from secreted molecules produced by glial cells and neurons in the assembly of transmitter and effector receptors and ion channels which affect brain function and neuronal control. KS is a component of neuropils in white matter; however, its specific roles in the function of this tissue have yet to be determined. Dysfunctional KS‐mediated cell signaling, however, may predispose to the development of a number of neurological disorders.

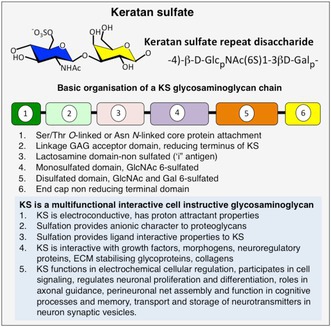

## Graphical abstract

Keratan sulfate (KS) is a multifunctional glycosaminoglycan with fascinating instructional regulatory, tissue organizational, and stabilization properties in the brain. KS has essential properties that regulate neuron proliferation and differentiation with synaptic roles and perineuronal net activities in cognitive activity and memory that define brain functional properties.

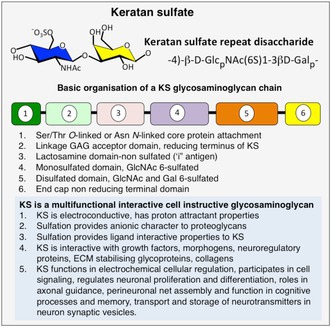

**Abbreviations**
ACAN: aggrecan
ALS: amyotrophic lateral sclerosis
BMPs: bone morphogenetic proteins
CA3: cornu ammonis region
Cdk4: cyclin‐dependent kinase 4
CHST2: GlcNAc6 sulfotransferase 1
CHST4: GlcNAc6 sulfotransferase 2
CHST5: GlcNAc6 sulfotransferase 3
CHST6: GlcNAc6 sulfotransferase 5
CHST7: GlcNAc6 sulfotransferase 4
CNS/PNS: central/peripheral nervous system
CS: chondroitin sulfate
ECM: extracellular matrix
ELISA: enzyme linked immunosorbent assay
FGF: fibroblast growth factor
FMOD: fibromodulin
GAG: glycosaminoglycan
HNK: human natural killer
IGFBP2: insulin‐like binding protein 2
iGnT: enzyme that selectively elongates the linear poly‐N‐acetyllactosamine region (“i” antigen) of KS
KERA: keratocan
KS: keratan sulfate
LUM: lumican
MAb 1B4: low sulfation KS monoclonal antibody
MAb 310G: low sulfation KS monoclonal antibody
MAb 5D4: high sulfation KS monoclonal antibody
MAb MZ14: high sulfation KS monoclonal antibody
MAP1B: microtubule associated protein‐1B
MUC16: ovarian cancer‐related tumor marker CA125
PNNs: perineuronal nets
PODXL: podocalyxcin
PRELP: proline/arginine‐rich end leucine‐rich repeat protein
RPTP‐ζ, RPTPZ: receptor protein tyrosine phosphatase zeta
Shh: sonic hedgehog
SLRP: small leucine rich proteoglycan
SOD: superoxide dismutase
SOD1(G93A): SOD1 G93A ALS mouse model
SV2: synovial vesicle 2 proteoglycan
TEM: transmission electron mictoscopy
Wnt: wingless‐integrated cell signaling pathway
β3GnT‐1‐7: β3N‐acetyl glucoaminyltransferases (1–7)
β4GalTs: β1,4‐galactosyltransferases

## 1 Introduction

**FIGURE 1:**
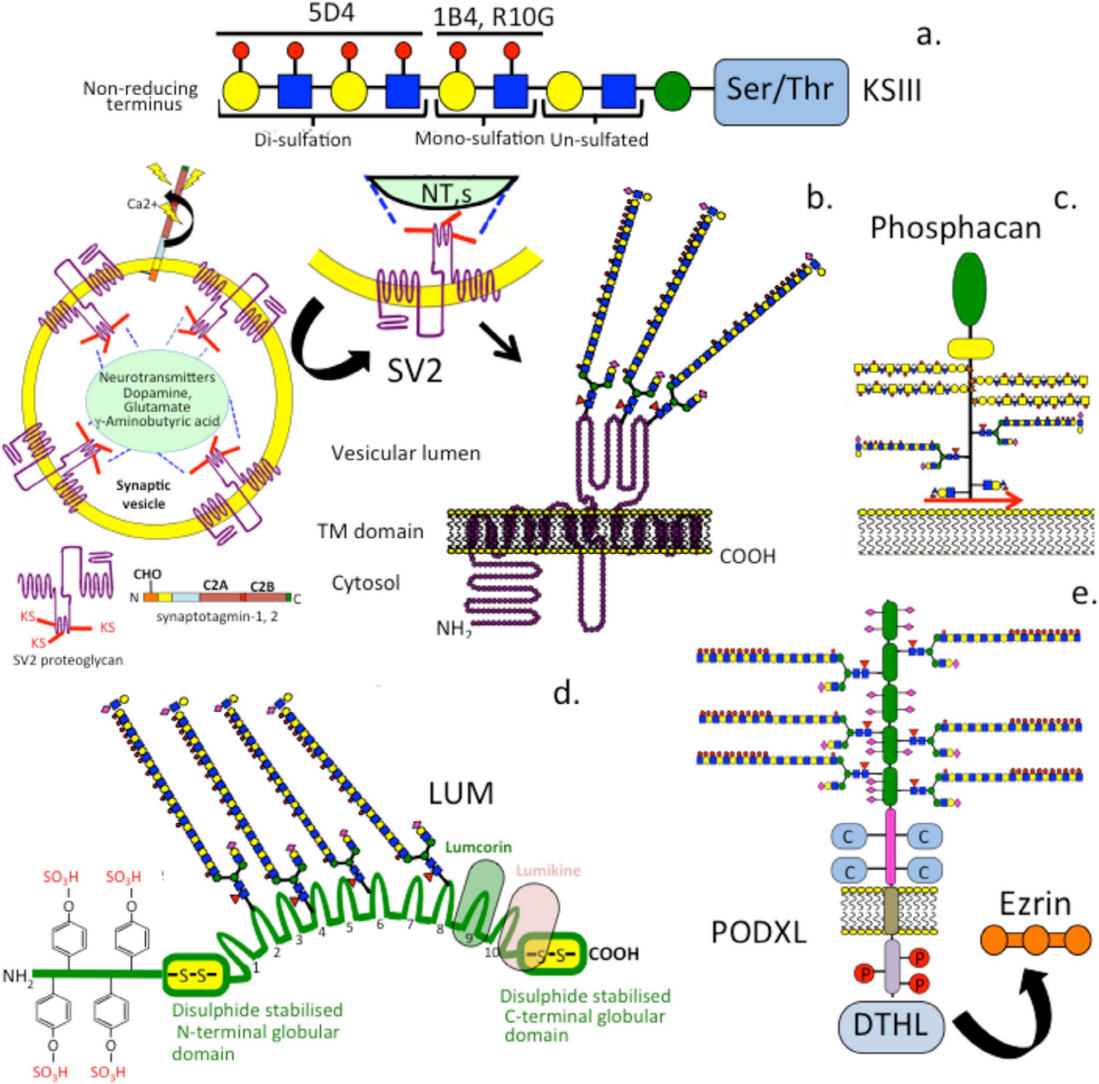
Brain keratan sulfate (KS) glycosaminoglycan chains are organized into di‐ and mono‐ sulfated regions identified by monoclonal antibodies 5D4, and 1B4, R10G (a). Examples of diverse forms of KS‐brain proteoglycans. SV2 PG, a 12 span transmembrane synaptic vesicle neurotransmitter (NT) transporter proteoglycan (b). Synaptotagmin interacts with SV2 coordinating transport of synaptic vesicles to the synaptic gap where these merge with the presynaptic membrane and NTs are released into the synaptic gap where they are taken up by communicating neurons in the network (b). Phosphacan is a major brain KS‐proteoglycan isoform which regulates neuronal activity and axonal migration and is a functional component of perineuronal nets and cognitive processes. The red arrow depicts endogenous protease activity that releases the phosphacan ectodomain from the cell surface where it resides as protein tyrosine phosphatase receptor–zeta (c). Lumican (LUM) is a multifunctional small leucine rich proteoglycan (SLRP) with ten highly interactive leucine rich repeat modules (LRRs), that regulate small collagen fibril assembly essential for vision. LRR9 is an MMP inhibitor module, a C‐terminal domain contains growth factor activity (lumikine) that regulates cell proliferation. N‐terminal sulfated tyrosine residues convey HS like interactivity in LUM (d). Podocalyxcin (PODXL) is an anti adhesive transmembrane sialo‐KS‐PG that regulates embryonic neuronal development, a DHTL (AspThr‐His‐Leu) peptide module interacts with the cytoskeletal protein Ezrin and regulates cell signaling (e).

Keratan sulfate (KS) was first identified almost 100 years ago and its basic structure elucidated (Funderburgh 2000, 2002). Development of KS specific endoglycosidases in the 1970s and a range of monoclonal antibodies in the 1980s significantly aided in the development of sensitive detection methods for KS (Caterson et al. 1983; Mehmet et al. 1986a). In this early KS work a major focus was on highly sulfated KS examined using monoclonal antibodies (MAbs) such as 5D4 and MZ14 (Caterson et al. 1983; Mehmet et al. 1986a). KS, however, has regions in the KS chain which are monosulphated and not detected by 5D4 and MZ14 antibodies (Figure 1) (Caterson and Melrose 2018). The highly sulfated regions on KS chains are predominantly towards the nonreducing terminus. More recently, antibodies to the low sulfated regions in KS have been developed (MAb 1B4, R10G) and the contributions of low sulfated KS regions to KS pathobiology are now emerging (Melrose 2019a, 2025a). KS is a multifunctional brain glycosaminoglycan (GAG) with essential roles in brain function and homeostasis (Melrose 2025a, 2019b, 2024a, 2024b, 2025b). Cues from the brain extracellular matrix (ECM) have instructive roles in the assembly and repair of functional networks from secreted molecules produced by glial cells and neurons in transmitter and effector receptors and ion channels which effect brain function and neuronal control (Melrose 2024a; Melrose et al. 2021).

## 2 Roles for Proteoglycans in Brain Functions

Brain proteoglycans (Figure 1) have diverse roles in biological interactions with growth factors, chemokines, morphogens, neural guidance molecules, factors that determine neural survival, and with ECM components that guide neural migration and formation of neural networks and perineuronal nets (PNNs) during synaptic development (Melrose et al. 2021; Sarnat and Yu 2025; Dityatev et al. 2010). KS interactions regulate development, neural proliferation, differentiation, and axonal guidance but may contribute to disease processes when these processes are dysfunctional (Melrose et al. 2021; Sarnat and Yu 2025; Schwartz and Domowicz 2023; Ennemoser et al. 2022; Wang and Chi 2022).

## 3 KS Biosynthesis

**FIGURE 2:**
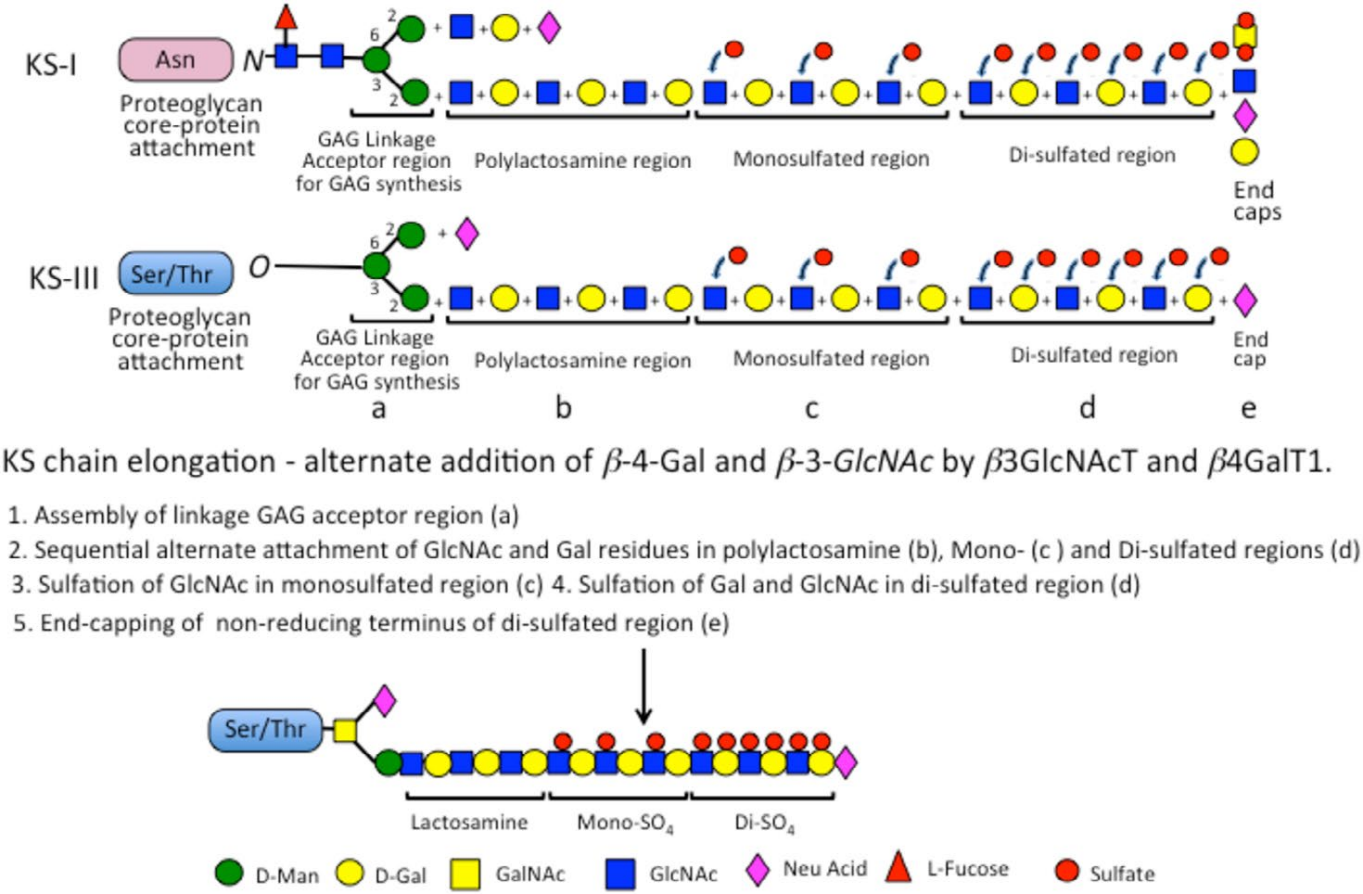
Schematic of the biosynthetic steps involved in corneal and brain keratan sulfate (KS), and different regions that have been identified along the KS glycosaminoglycan chain (a–e).

The polylactosamine backbone of KS is produced by two families of glycosyltransferases, β1,4‐galactosyltransferases (β4GalTs) and β1,3‐*N*‐acetylglucosaminyltransferases (β3GnTs). In human and mouse, there are 7 enzymes in the β4GalT family and 8 enzymes in the β3GnT family. Extension of KS chains occurs through glycosyltransferases that add *β*‐4‐galactose and *β*‐3‐*N*‐acetylglucosamine to the acceptor linkage region to extend the nascent KS chain (Ujita et al. 2000; Shiraishi et al. 2001). The polylactosamine region in KS is known as “i” antigen and attaches to the linkage glycosaminoglycan (GAG) acceptor region (Figure 2). During chick development, corneal *β*4Gal‐T increases with development, and corneal KS synthesis is maintained at high levels in adult corneal cells (Cai et al. 1996). Once the nascent KS chain is formed, it is then sulfated. Sulfation of the Gal and GlcNAc residues occurs at the 6‐O positions in the di‐sulfated region. Di‐sulfation only occurs in the nonreducing terminus after the GlcNAc residues are first sulfated. In monosulfated regions on KS, only the GlcNAc residues are sulfated. There are five enzymes (CHST2 (GlcNAc6ST1), CHST4 (GlcNAc6ST2), CHST5 (GlcNAc6ST3), CHST6 (GlcNAc6ST5), and CHST7 (GlcNAc6ST4)) that are responsible for GlcNAc‐sulfation in humans and four enzymes in mouse. CHST6 is the major enzyme responsible for KS sulfation in humans (Akama and Fukuda 2014). CHST1 (KSGal6ST) is the enzyme responsible for Gal sulfation in KS (Fukuta et al. 1997).

A number of proteins modified with linear poly‐*N*‐acetyllactosamine would be considered KS if they were sulfated (Hanisch et al. 1989).

## 4 KS Biosynthetic Enzymes

### 4.1 Galactosyltransferases

Glycosyltransferases are responsible for the sequential addition of Gal and GlcNAc to the growing KS chain (Funderburgh 2000). Several families of galactosyltransferases (GalT) have been identified (Amado et al. 1999). β4GalT1 catalyzes the addition of UDP‐Gal to a nonreducing terminal GlcNAc acceptor, via a β1–4 glycosidic linkage generating the non‐sulfated poly‐*N*‐acetyllactosamine domains which comprise the basic unit of KS. Galactosyltransferase, β4GalT4, is the only galactosyltransferase that catalyzes the transfer of Gal to a nonreducing terminal GlcNAc‐6‐sulfate acceptor residue and is essential for the production of mono‐ and disulfated regions in the KS chain. β4 GalT4 is also the only GalT enzyme which generates the initial branch points found in the 2 *O*‐linked poly‐*N*‐acetyllactosamine regions of the KS core structure. KS is the only branched GAG.

### 4.2 *N*‐Acetylglucosaminyltransferases

Seven β3N‐acetyl glucoaminyltransferases (β3GnT‐1, 2, 3, 4, 5, 6, 7) catalyze the addition of GlcNAc viaβ3 linkage to non‐reducing terminal Gal or GalNAc (Caterson and Melrose 2018). Of all of the KS degradative enzymes known, iGNti glucoaminyltransferase acts most efficiently in the elongation of the linear poly‐*N*‐acetyllactosamine region.

### 4.3 Sulfotransferases

Sulfotransferase enzymes transfer sulfate groups from the 3′‐phosphoadenosine 5′‐phosphosulfate (PAPS) donor to C6 of Gal or GlcNAc in KS; KSGal6ST transfers sulfate groups to C6 of Gal, and GlcNAc6ST transfers sulfate to the C6 of GlcNAc (Funderburgh 2000, 2002; Caterson and Melrose 2018). Five GlcNAc‐6‐sulfotransferase genes have been identified. GlcNAc6ST‐5 sulfates the nonreducing terminal GlcNAc in both human and mouse tissues; KSGal6ST catalyzes the addition of sulfate to an internal Gal residue within the KS chain (Fukuta et al. 1997). Sulfation of Gal in unsulfated KS disaccharides is much lower than in disaccharides where GlcNAc is already sulfated.

## 5 The Emergence of Low Sulfation KS Epitopes in Cellular Regulation

The aggrecan and protein tyrosine phosphatase receptor zeta (phosphacan) linkage regions are GAG acceptor modules for KS biosynthesis in cartilage and brain (Funderburgh 2002; NarentuyaTakeda‐Uchimura et al. 2019). KS chains in the cornea are longer and more sulfated than cartilage KS. Highly sulfated regions at the nonreducing terminus occur in brain KS. Despite its misnaming, corneal KS found in lumican, fibromodulin, keratocan, and PRELP in brain tissues contain small low sulfated N‐linked KS isoforms (Melrose 2019b). A low sulfation mucin‐like KS glycoconjugate in elasmobranch fish has electroconductive properties and detects prey fish species by electrolocation through the electric fields they emit from muscular exertion (Melrose 2019a). Mucin‐like glycopolymer gels in electrosensory tissues generate cues that direct electrolocation in amphibians and neuronal activation in mammals (Melrose 2019c). Two terrestrial monotreme species, the duck‐billed platypus and echidna, are the only land‐based animals that have retained the ability to electrolocate (Melrose 2019a).

KS is an instructive GAG that regulates neuronal control of specific brain functional properties (Melrose 2024b, 2025b). KS‐proteoglycans play important roles in regulating the development and functions of the brain (Melrose 2019b; Hayes and Glycans 2018; Nishiwaki et al. 1998). Spatial and temporal regulation of an isoform of Phosphacan/RPTP‐zeta that contains a low sulfation RG10 positive KS epitope appears critical for critical period plasticity (Takeda‐Uchimura et al. 2015). Modification of the structure of phosphacan/RPTP‐zeta modifies its interactive properties with cells, providing diverse roles in neuronal regulation, axonal guidance, and the architecture and function of PNNs, effecting cognitive processes, memory, and critical period plasticity in the visual cortex (Takeda‐Uchimura et al. 2015; Eill et al. 2020; Faissner et al. 2006; Sinha et al. 2023; Hamanaka et al. 1997).

Immunohistochemical analyses have demonstrated the presence of KS containing disaccharide units of N‐acetyl GlcNAc‐6‐sulfate and nonsulfated galactose during critical period plasticity in the visual cortex; however, KS containing disaccharide units of GlcNAc‐6‐sulfate and galactose‐6‐sulfate were already known to disappear before that period, showing that dynamic remodeling of glycan structure has functional impacts in brain tissues. KS chains were distributed diffusely in the extracellular space and densely around the soma of a large population of excitatory and inhibitory neurons. Electron microscopic analysis revealed KS localized within the perisynaptic spaces and in dendrites but not in presynaptic locations (Takeda‐Uchimura et al. 2015). KS‐phosphacan with a specific sulfation pattern is necessary for the generation of long‐term potentiation in the critical period to modulate neuronal activities in brain tissues as part of its cell regulatory arsenal.

In the mouse brain, GlcNAc6ST is required for the synthesis of GlcNAc‐6‐sulfated KS chains that have roles in neuronal plasticity in the visual cortex during the critical period, but not in adulthood (NarentuyaTakeda‐Uchimura et al. 2019). A critical period is a maturational developmental stage where the nervous system is exposed to environmental stimulation. GlcNAc‐6‐sulfated MAb R‐10G positive KS proteoglycans are found diffusely in neuropils and densely in PNNs in the adult visual cortex. GlcNAc6ST3, an intestinal GlcNAc6ST, is a major brain oligodendrocyte KS sulfotransferase in adults. An isoform of the protein‐tyrosine phosphatase PTPRZ is an R‐10G positive KS proteoglycan with functional roles that are being investigated for their impacts in critical periods in brain development.

Protein‐tyrosine phosphatase receptor type z1 (Ptprz1)/phosphacan forms a KS scaffold in the brain which is extended by Beta3Gn‐T7. This is a major KS biosynthetic enzyme in neuropils and the perineuronal region in the adult brain. The *B3gnt7* gene is selectively expressed in oligodendrocyte precursor cells (OPCs) and oligodendrocytes and, along with GlcNAc6ST3, appears to play a role in the formation of neuropils and perineuronal nets in the adult brain through the synthesis of R‐10G‐positive KS proteoglycan (Takeda‐Uchimura et al. 2022). The neuropil is an intertwined network of axons, dendrites, and glial cells in the CNS gray matter ECM.

## 6 KS Functional Roles in Brain Tissues

KS is an abundant brain GAG, the second richest tissue source of KS after the cornea. KS has important roles in cellular regulation and has instructive roles in neurodevelopmental processes (Melrose 2019b, 2024a, 2025b; Hayes and Glycans 2018) and unique functional aspects of this GAG continue to be uncovered (Caterson and Melrose 2018; Melrose 2019a, 2024b, 2019c). Information contained within the sulfated sugars and poly‐*N‐*acetyllactosamine regions of KS promotes growth factor and morphogen binding. Interactions with fibroblast growth factor (FGF), insulin‐like growth factor binding protein‐2 (IGFBP2), wingless‐type mouse mammary tumor virus, integration site family morphogen (Wnt), sonic hedgehog (Shh), and bone morphogenetic proteins (BMPs) regulate essential brain physiological and developmental processes (Russo et al. 1997; Weyers et al. 2013). The interaction of KS with IGFBP2 may be significant (Caterson and Melrose 2018). The activity of insulin‐like growth factors through their receptors in intracellular signal pathways is modulated by the IGF binding proteins (Yau et al. 2015). KS thus may further modulate the activity of the IGFBPs, providing an additional level of control over cellular activity. The cyclin‐dependent kinase 4 (Cdk4) pathway is operative in neuritogenesis and is activated by insulin, inducing neural cell proliferation and terminal differentiation (Chirivella et al. 2017). Furthermore, microarray studies with corneal KS have also shown it interacts with the Ephrin 4 receptor, promoting neuronal cell proliferation and differentiation through Ephrin tyrosine kinase activity (Liu et al. 2017). A number of cell‐associated and extracellular KS‐proteoglycans have been identified in brain tissues (Table 1) with a diverse range of functional properties that control neural development, neurotransduction, neural protection, cognition and memory, cellular proliferation and differentiation, and the maintenance of tissue homeostasis (Melrose 2025a, 2019b, 2024a). Cues from the brain ECM have instructive roles in the assembly and repair of functional network structures from secreted molecules produced by glial cells and neurons that are used to assemble transmitter and effector receptors and ion channels, which affect brain function and neuronal control (Melrose et al. 2021).

**TABLE 1:** Examples of the diverse forms and functions of brain KS‐proteoglycans.

PG	Features	Mw	GAG
Phosphacan	Soluble ectodomain of RPTP‐zeta, has roles in perineuronal net assembly and function, cognitive processes, memory. Modulates neurite extension in neural networks (Faissner et al. 2006; Maurel et al. 1994; Garwood et al. 1999; Garwood et al. 2003; Fujikawa et al. 2017).	300	KS, CS, HNK‐1
Aggrecan (ACAN)	Space‐filling proteoglycan that along with HA compartmentalizes and hydrates brain tissues. Major component of perineuronal nets, neuroprotective (Morawski et al. 2012). HNK‐1 trisaccharide in brain aggrecan provides cell interactive properties (Yabuno et al. 2015).	208–220	CS, KS, HNK‐1
Synaptic vesicle PG (SV2)	Synaptic vesicle neurotransmitter transporter and smart storage PG, SV2A, SV2B, SV2C paralogs share 60% sequence and 80% structural homology. SV2A controls neurotransmitter release (Nowack et al. 2010; Wan et al. 2010), SV2B is expressed in the retina (Morgans et al. 2009), SV2C modulates dopamine release and is disrupted in Parkinson disease (Dunn et al. 2017).	250 and 100	KS
Podocalyxcin (PODXL)	Transmembrane, anti‐adhesive sialo‐KS‐proteoglycan, upregulated in many cancers (Le Tran et al. 2021; Toyoda et al. 2017a, 2017b). May contain high and low sulfation KS chains. Has multiple roles in neural development and synapse formation (Vitureira et al. 2005; Vitureira, Andres, et al. 2010).	65	KS
Lumican (LUM)	Lumican isa small leucine rich repeat proteoglycan (SLRP) multifunctional KS‐proteoglycan. Leucine rich repeat domain‐9 (LRR9) is a peptide module (lumcorin) and a matrix metalloprotease (MMP) inhibitor (Zeltz et al. 2009; Pietraszek et al. 2014). LUM has anti‐angiogenic (Niewiarowska et al. 2011) and anti‐tumor properties (Zeltz et al. 2009; Coulson‐Thomas et al. 2013; Pietraszek et al. 2013). LUM regulates synthesis of small regularly spaced collagen fibrils in cornea essential for vision (Nikitovic et al. 2008; Kao and Liu 2002).	38	KSI
Fibromodulin (FMOD)	Fibromodulin is a regulatory multifunctional matricellular proteoglycan with roles in cellular architecture and tissue function, regulates large collagen fiber fibrillogenesis, binds C1q, activates the Complement pathway (Zheng et al. 2023; Jan et al. 2016; Al‐Qattan and Al‐Qattan 2018; Sjoberg et al. 2005; Svensson et al. 2000).	42	KSI
Keratocan (KERA)	Essential developmentally regulated ECM component of the lens capsule, organizes collagen fiber diameters and spacing in the corneal stroma to maintain stromal clarity (Puri et al. 2020; Gesteira et al. 2023; Matsushima et al. 2019; Stepp and Menko 2024).	37–50	KSI
Claustrin (MAP1B)	Claustrin is an anti‐adhesive neural proteoglycan (Burg et al. 1994) which inhibits neurite outgrowth, related to MAP‐1B (Edelmann et al. 1996). MAP1B is a 225 kDa microtubule and dendritic process neuron and glial cell proteoglycan.	225	KS

## 7 KS Is a Functional Component in Biodiverse Brain Proteoglycans

The KS structure based on poly‐N‐acetyl‐lactosamine‐Galβ(1‐4)GlcNAcβ(1‐3)‐ is 6‐*O*‐sulfated on GlcNAc and Gal in di‐ and mono‐sulfated regions in the KS chain and is non‐sulfated in polylactosamine regions (Figure 2). The aforementioned KS chain regions vary temporally and spatially in size in specific tissue contexts and with aging, providing a significant level of structural heterogeneity and variable functional capability in KS (Funderburgh 2000; Caterson and Melrose 2018). Three forms of KS have been identified, namely corneal KSI, skeletal KSII, and brain KSIII. KS‐I GAG chains are linked to protein cores via an N‐linked asparagine residue in fibromodulin, lumican, and keratocan. KS‐II chains are *O*‐linked to serine or threonine residues in aggrecan. KS‐III chains are attached to core protein serine or threonine via mannose residues. KS‐III is abundant in brain proteoglycans such as claustrin, phosphacan, and SV2 (Table 1). SV2 is a 12 span transmembrane KS‐proteoglycan that occurs as 3 alternatively spliced isoforms (SV2A, B, C) differing in tissue localization. SV2 functions in the storage and transport of synaptic vesicle neurotransmitters (Bajjalieh et al. 1992; Nowack et al. 2010) and interacts with the acetylcholine transporter receptor vesamicol and synaptotagmin, controlling the availability of acetylcholine and ATP released from synaptic vesicles (Caterson and Melrose 2018). SV2 is also complexed with α5 laminin on the nerve terminal surface, facilitating synaptic interactions (Son et al. 2000). Phosphacan, the exodomain of RPTP‐ζ, can be substituted with CS, KS, or HNK‐1 trisaccharide, which conveys diverse interactive properties and the ability to influence neuritogenesis (Maurel et al. 1994). The KS chains of phosphacan provide anti‐adhesive cues over neuronal cells, preventing their interaction with tenascin‐C; this promotes neurite outgrowth following injury in axonal repair (Dobbertin et al. 2003). Phosphacan also has important architectural roles in the assembly and function of perineuronal nets (Takeda‐Uchimura et al. 2015; Eill et al. 2020; Sinha et al. 2023). Furthermore, a specific sulfation pattern in phosphacan KS promotes critical period plasticity in the visual cortex (Takeda‐Uchimura et al. 2015). A critical period is a developmental window where environmental input cues drive the appropriate development of relevant brain circuits (Cisneros‐Franco et al. 2020). KS has important roles to play in critical periods of plastic changes in ocular tissues. Beta3Gn‐T7, a keratan sulfate β1, 3 N‐acetylglucosaminyltransferase, is involved in the biosynthesis of the lactosamine repeats in KS (Takeda‐Uchimura et al. 2022); GlcNAc6ST3 is a KS sulfo‐transferase that regulates PTPRZ structure and functional diversity (NarentuyaTakeda‐Uchimura et al. 2019).

## 8 Emerging Roles for KS Glycans in the Pathobiology of Brain Tissues

GlcNAc6ST3 synthesizes R‐10G‐positive KS epitopes (a dimeric asialo 6‐sulfo lactosamine) in the brain. KS oligosaccharides have been proposed to stimulate mesothelial cell proliferation and differentiation and may have similar activity in the brain. KS oligosaccharides in mammalian milk are an important nutritional resource and have potential modulatory properties optimizing brain development in early life (Fan et al. 2023). 2′‐Fucosyllactose is a major oligosaccharide found in human breast milk that may promote development of cognitive processes in mice through modulatory effects on the gut microbiota, enhancing 5‐hydroxytryptamine levels in the brain through the gut–brain axis (Zhu et al. 2025). The lactosamine domains of KS could potentially be released by endogenous protease activity and may have neuroregulatory activity. MUC16 has been proposed as a candidate carrier protein for pleural R‐10G‐reactive KS glycans; mucins may have similar roles in brain tissues (Takeda‐Uchimura et al. 2024). Mucin‐like glycopolymer gels in electrosensory tissues generate neuroregulatory cues (Melrose 2019c). A KS disaccharide (L4, [SO_3_^−^‐6]Galβ1‐4[SO_3_^−^‐6]GlcNAc) has also been shown to significantly attenuate alveolar destruction, reducing the influx of neutrophils, levels of inflammatory cytokines, and tissue‐degrading MMPs and myeloperoxidase in a mouse elastase‐induced emphysema model. L4 represents a potential drug for the treatment of COPD, facilitated by its anti‐inflammatory properties but also is of relevance to neuroinflammation (Gao et al. 2017). Furthermore, during embryonic development, highly sulfated KS is detected in the developing notochord and otic vesicles and has been used as a notochordal molecular marker. The KS chain‐synthesizing genes, beta‐1,3‐N‐acetyl glucosaminyltransferase (*b3gnt7*) and beta‐1,4‐galactosyltransferase (*b4galt4*), are strongly expressed in the notochord (Yasuoka 2023). Histochemical and immunoelectron microscopy has demonstrated 5D4 positive KS modified with sialic acid and fucose in Mac2/galectin‐3‐positive activated or proliferating microglia in an Amyotrophic lateral sclerosis (ALS) mouse model. Galactose‐6‐ and GlcNAc‐6S in the spinal cord and brainstem are associated with spinal degeneration and pathological bulbar lower motoneurons. KS glycans may activate microglial cells and play a role in ALS disease progression (Foyez et al. 2015). The role of KS in ALS motoneuron degenerative disease has been examined using the ALS model SOD1(G93A) and KS deficient GlcNAc6ST‐1(−/−) mice (Hirano et al. 2013). KS expression was induced exclusively in a subpopulation of microglia in SOD1(G93A) mice surrounding motoneurons in the ventral horn during the early ALS disease phase when M2 microglia markers were transiently enhanced in SOD1(G93A) mice, but not in SOD1(G93A)GlcNAc6ST‐1(−/−) mice. KS expression in microglia has also been reported in some human ALS cases. KS may have a powerful, suppressive role in the early pathogenesis of ALS, representing a novel therapeutic target. KSPGs inhibit nerve regeneration after CNS injury. Induction of autoimmune encephalomyelitis in the KS‐KO mouse deficient in the N‐acetylglucosamine (GlcNAc)‐6*‐O*‐sulfotransferase 1 (GlcNAc6ST1) gene develops less severe encephalomyelitis (Ueno et al. 2015). GlcNAc6ST1 may have roles in the passage of pathogenic lymphocytes through the blood–brain barrier (BBB); KS may thus modulate this process and be useful in the treatment of neuroimmunological diseases. Following traumatic brain injury, GlcNAc 6‐O‐ST‐1 is required for brain KS biosynthesis and glial scar formation as part of a tissue stabilization protective response to a traumatic defect. Such glial scars also inhibit neural outgrowth and repair processes, and this represents a negative aspect to KS pathobiology (Zhang, Muramatsu, et al. 2006; Zhang, Uchimura, and Kadomatsu 2006). There are no doubts that roles for KSPGs and KS glycans in brain pathobiology will continue to be discovered, emphasizing the regulatory functions of KS in brain and related tissues.

## 9 KS Has Roles in Tumors

While KS is an important component of the ECM of normal brain tissues, it is also a component of the tumor environment (Hayes and Melrose 2020a). Neural crest cell peripheral neuroblastic tumors frequently occur in neuroblastomas, ganglioneuroblastomas, and ganglioneuroma solid neoplasms of the CNS/PNS (Shayestehfar et al. 2025). Malignant astrocytic tumors also display an elevated expression of highly sulfated KS (Kato et al. 2008). Podocalyxcin is an anti‐adhesive transmembrane polysialylated‐KS proteoglycan with essential roles to play in normal neural development (Vitureira et al. 2005; Vitureira, Andrés, et al. 2010) and is produced by human embryonic and induced pluripotent stem cells (Toyoda et al. 2017b). Podocalyxcin is upregulated in glioblastoma and in astrocytomas (Kato et al. 2008; Binder et al. 2013; Hayatsu, Kaneko, et al. 2008; Hayatsu, Ogasawara, et al. 2008; He et al. 2010; Liu et al. 2015, 2014), and is a prognostic biomarker for several cancers (Nielsen and McNagny 2009; Wang et al. 2017). The sulfation of the KS chains on podocalyxcin on normal embryonic cells and tumor cells differs. The former express a low sulfation KS isoform detected by MAb R‐10G (Kawabe et al. 2013; Makanga et al. 2015; Nakao et al. 2017), tumor cells, however, produce a high sulfation KS isoform (Hayatsu, Kaneko, et al. 2008) detected by antibodies 5‐D‐4, MZ‐14, or 4C4 (Caterson et al. 1983; Fukuma et al. 2003; Mehmet et al. 1986b). Highly sulfated KS is prominent in carcinomas of the genital tract (Miyamoto et al. 2011), prostatic secretory cells (Cohen et al. 2000), brain and ovarian tumors (Whitham et al. 1999), papillary carcinomas of the human thyroid gland (Ito et al. 1996), and granular cell tumors (Ehara and Katsuyama 1990), malignant astrocytic tumors (Kato et al. 2008; Hayatsu, Kaneko, et al. 2008), and glioblastomas (Hayatsu, Ogasawara, et al. 2008). The diverse and unique sulfation sequences of KS decorating tissue proteoglycans significantly impact cellular regulation through variable critical effects on the activity of cellular mediators such as cytokines and growth factors and neuroregulatory proteins in neural network development and neuron function. When KS‐mediated cell signaling is dysfunctional, this leads to the onset of disease processes (Caterson and Melrose 2018; Melrose 2019a, 2025a, 2024a, 2024b, 2025b; Hayes and Glycans 2018).

## 10 KS Has Roles in Tissue Structural Organization and Functional Properties

**FIGURE 3:**
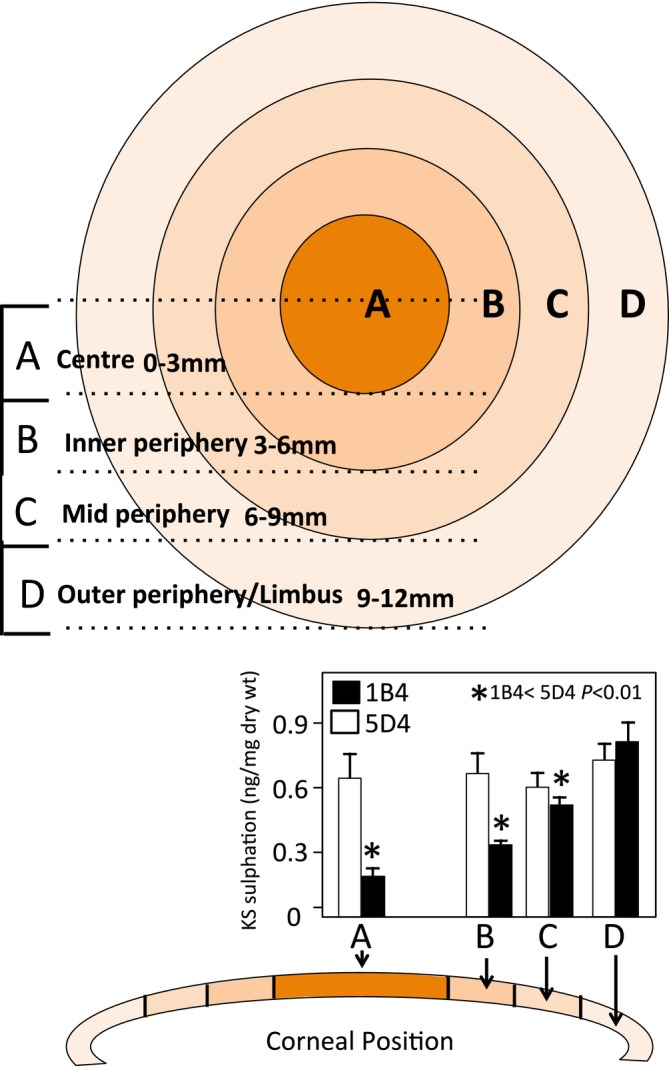
Schematic depiction of a cornea and zonal locations with variable high (5D4) and low (1B4) sulfation keratan sulfate epitopes. Figure reproduced from (Melrose 2019a) open access.

The cornea displays high and low‐sulfated KS epitopes demonstrated by immunohistochemistry and ELISA that have been correlated with collagen fibril architecture in central to peripheral corneal regions (Young et al. 2007; Quantock et al. 2010; Ho et al. 2014). Electron microscopy and X‐ray fiber diffraction were used to assess collagen fibril architecture. Small angle X‐ray diffraction and transmission electron microscopy showed collagen fibril diameters remain relatively constant with regularly spaced small collagen fibers centrally until the outer periphery of the cornea was reached, where fibrils became more widely spaced and of larger diameter as the sclera was approached. Depth‐profiled synchrotron microbeam analyses showed fibril diameters were greater superficially than in deeper regions from the corneal center outwards. Low sulfation KS increased towards the cornea periphery, with its levels lowest in the central region of the cornea, while highly sulfated KS levels were relatively constant throughout the cornea (Figure 3). Lumican and fibromodulin have roles in collagen fibrillogenesis, with the former regulating production of small regularly spaced collagen fibers essential for vision, while fibromodulin regulates production of large collagen fibers, which provide mechanical stability (Kao and Liu 2002; Chen et al. 2014, 2010; Chakravarti 2002).

In an unrelated study, high and low sulfation KS epitopes have been immunolocalised in defined centres in brain tissues in so‐called brain song centres with roles in song‐learning in the juvenile male zebra song‐finch (Fujimoto et al. 2015). The zebra song finch model has provided valuable insights into brain KS functionality and has now been developed into models that are being used to examine human language acquisition processes and the investigation of the neurobiological basis of vocal learning (Melrose 2025a; Spierings and Ten Cate 2016; Mello 2014).

## 11 Astrocyte and Microglial KS

Astrocytes and microglial cells play vital roles in the regulation of CNS neural patterning during development and following injury, support neurite extension, and express KS‐proteoglycans with axonal guidance roles (Powell et al. 1997; Reddaway et al. 2023). Microglia are primary CNS immune cells with properties similar to macrophages in terms of how they respond to pathogens and traumatically injured brain tissues (Colonna and Butovsky 2017; Thompson and Tsirka 2017). Microglia migrate to sites of infection or injury, where they destroy pathogens or remove damaged tissue components. Microglial cells in the developing CNS regulate brain development, have roles in the maintenance of neuronal networks, and in the repair of damaged brain tissues through their ability to clear cell debris and regulate misdirected or transitory axons, supporting cell survival and neurite outgrowth (Gao et al. 2023). Microglia secrete cytokines that regulate CNS development through distinct effects on different neural cell types (Michell‐Robinsonm et al. 2015; Yu et al. 2022). Microglia are a heterogeneous cell type (Reddaway et al. 2023), 5D4 positive and 5D4‐negative KS stained microglia have been identified in the rat brain hippocampus. Ramified 5D4 positive microglia numbers are higher in the *Stratum Oriens* of the CA3 (Cornu Ammonis) region, a region that has been referred to as the “pacemaker” of the hippocampus, a critical region in the temporal lobe of the brain involved in memory formation, spatial navigation, and other higher‐order cognitive functions. 5D4 KS positive microglia have a high number of cellular processes, which provides them with increased interactive capability with brain tissue components and may represent a unique subtype associated with synapses. Mossy fiber synaptic connections in CA3 pyramidal neurons have been noted to be disrupted in the hippocampus of schizophrenics; this likely contributes to the higher cognitive function and brain dysfunction described in this condition (Kolomeets et al. 2005). Microglia also have critical roles in neuroinflammatory responses and homeostatic brain regulation by modulating synapse formation, pruning, and tissue repair responses (Thompson and Tsirka 2017). Phosphacan regulates mossy fiber outgrowth and regeneration; thus, it has potential roles to play in beneficial tissue remodeling with microglia, which may have functional consequences (Butler et al. 2004).

## 12 Specific Organizational and Structural Roles for Brain KS‐Proteoglycans

KS‐proteoglycans have tissue organizational and axonal guidance roles in brain development that determine brain function (Melrose et al. 2021; Sarnat and Yu 2025; Schwartz and Domowicz 2023; Saied‐Santiago and Bülow 2018). Cell‐ECM communication is important in cellular regulation and cell migration (Sarnat and Yu 2025). ECM components such as GAGs are important sources of information with instructive roles to play in cellular regulation (Melrose et al. 2021). The brain ECM is a highly dynamic environment that plays a critical role in neural excitation, signal transmission, development, aging, and neurological disorders (Zhang et al. 2025). The interconnectivity and structural organization of brain tissues are thus important considerations with regard to brain function (Schuz and Braitenberg 2002; Nieuwenhuys et al. 1988; Ouyang et al. 2017; Catani et al. 2012). Dysregulation of KS‐proteoglycans may result in cerebral dysgenesis, a brain malformation characterized by incomplete development, abnormal growth, incomplete brain division, or incomplete organization of the developing brain (Sarnat and Flores‐Sarnat 2024).

## 13 Brain Tissue Structural Connectivity and Functional Impacts

Macro interconnected anatomical cortical and white matter fiber networks support cellular communication and coordinated functional properties in the brain (Friston 2002). KS‐proteoglycans and the ECM direct the development of such structures and are components of neuropils; neuropilins lock secreted semaphorins onto plexins in a ternary signaling complex (Janssen et al. 2012), instructive cues delivered by the ECM to brain cell populations regulate cellular activity and maintain the functionality of the mature brain (Melrose 2019b, 2024a; Melrose et al. 2021; Dzyubenko and Hermann 2025). Cognitive functional and adaptive properties of the brain rely on the structural dynamics of such large‐scale assembled networks (Litwińczuk et al. 2023). KS has been demonstrated as a component of white matter; however, its roles in the structural organization and function of this tissue are not known (Janssen et al. 2012). The *B3gnt7* gene is selectively expressed in oligodendrocyte precursor cells (OPCs) and oligodendrocytes and, along with GlcNAc6ST3, appears to play a role in the formation of neuropils and perineuronal nets in the adult brain through the synthesis of R‐10G‐positive low sulfation KS‐proteoglycans (Takeda‐Uchimura et al. 2022).

Diffusion magnetic resonance imaging (dMRI) tractography, an advanced imaging technique has enabled in vivo reconstruction of the brain's white matter interconnectivity at macro scale and is an important tool for quantitative mapping of the brain's microstructure (Zhang et al. 2022). This technique has demonstrated the crucial role the *Corpus Collosum*, the largest compact white matter fiber bundle of the human brain involved in interhemispheric transfer, plays in bipolar pathophysiology, cognitive impairment and motor dysfunction that characterizes this psychiatric disorder (Videtta et al. 2023). Short association U‐fibers are implicated in neurological and psychiatric diseases. dMRI may provide insights into the role of KS‐proteoglycans in brain tissues in the future. MRI and diffusion tensor imaging (DTI) has demonstrated abnormalities in U‐fiber regions in autism spectrum disorder (Sundaram et al. 2008; Patil et al. 2024), schizophrenia (Phillips et al. 2011; Nazeri et al. 2013), and ad (Phillips et al. 2016), KS‐proteoglycans may well have important roles to play in functional disorders which have yet to be elucidated. KS is highly interactive with a diverse range of ECM components, growth factors, morphogens, and neuroregulatory proteins (Melrose 2019b; Conrad et al. 2010) and has electroconductive properties that allow it to participate in neuroregulatory cell signaling (Melrose 2025a, 2024b, 2025b; Hayes and Glycans 2018), synaptic interactions and cognitive processing in PNNs (Takeda‐Uchimura et al. 2015; Eill et al. 2020; Sinha et al. 2023; Maurel et al. 1994; Dobbertin et al. 2003; Dino et al. 2006; Maeda and Noda 1996).

## 14 Cell‐ECM Interactions in Brain Tissues and Cellular Regulation

Cell‐ECM interactions, provide a highly regulated dynamic environment for neuroglial and astrocyte communication. ECM proteoglycans and GAGs are a vital molecular recognition, information transfer and storage system and have cell instructive roles in brain development and in the maintenance of homeostatic brain function throughout life (Dityatev et al. 2010; Dzyubenko and Hermann 2025). PNNs have important protective and instructive regulatory properties over the neuron populations they surround. PNNs are a dense form of ECM surrounding the soma and dendrites of neurons with roles in critical periods of developmental plasticity (Fawcett et al. 2019). The ECM provides instructive cues during the development and function of brain tissues (Melrose 2025a, 2019b, 2024a, 2025b, 2023; Melrose et al. 2021; Hayes and Glycans 2018; Hayes and Melrose 2020b, 2021, 2023). The perinodal ECM that surrounds the axonal nodes of Ranvier appear as myelination reaches completion, acting as ion‐diffusion barriers that affect axonal conduction speed (Bekku and Oohashi 2019). PNNs control CNS plasticity and promote functional recovery after a variety of CNS lesions and have roles in the regulation of memory (Fawcett et al. 2022) as has been shown in a number of psychiatric disorders (Carceller et al. 2023). PNNs play prominent roles in early neural development, migration of neurons and axonal path‐finding. PNNs also have important roles in the provision of synaptic plasticity to brain tissues (John et al. 2022).

## 15 Conclusion

KS has diverse, interactive properties as a brain ECM and cellular component and has unique cell regulatory properties of importance in normal brain functional properties. Disturbance of the normal organization of brain tissues and resultant dysfunctional GAG and proteoglycan activities likely contributes to development of neuropsychiatric disorders, and points to the important regulatory roles played by the brain ECM in normal brain function (Melrose 2019a, 2025a, 2019b, 2024a, 2024b, 2025b, 2019c, 2023; Melrose et al. 2021; Dityatev et al. 2010). A thorough understanding of the roles of ECM components in brain function may aid in the development of more effective GAG‐mediated therapeutic procedures for the treatment of psychiatric conditions. While KS is already known to display novel functional capability in the brain much correlative work still needs to be undertaken to link this to the structural reorganization of brain tissues known to occur in psychiatric disorders and show how this effects brain function.

## Author Contributions

**James Melrose:** conceptualization, funding acquisition, writing – original draft, writing – review and editing.

## Disclosure

J.M. has received consultancy fees from Arthropharm‐Fidia Pharmaceutics, Sydney. This company had no input into the content or interpretation of data presented in this review or the reason to publish.

## Conflicts of Interest

The author declares no conflicts of interest.

## Peer Review

The peer review history for this article is available at https://www.webofscience.com/api/gateway/wos/peer‐review/10.1111/jnc.70208.

## Data Availability

The data that support the findings of this study are available on request from the corresponding author. The data are not publicly available due to privacy or ethical restrictions.
